# Predictors of major infections in systemic lupus erythematosus

**DOI:** 10.1186/ar2764

**Published:** 2009-07-15

**Authors:** Guillermo Ruiz-Irastorza, Nerea Olivares, Ioana Ruiz-Arruza, Agustin Martinez-Berriotxoa, Maria-Victoria Egurbide, Ciriaco Aguirre

**Affiliations:** 1 Department of Internal Medicine, Hospital de Cruces, University of the Basque Country, Pza Cruces s/n, 48903 Barakaldo, Bizkaia, Spain

## Abstract

**Introduction:**

Infections commonly complicate the course of systemic lupus erythematosus (SLE). Our aim is to investigate the clinical predictors of major infections in patients with SLE.

**Methods:**

A nested case–control study design was used within the prospective Lupus-Cruces cohort. The endpoints of the study were major infections. Cases were defined as patients with a major infection. Two controls (SLE patients without major infections), matched for time of follow-up until the event and age at diagnosis, were selected for each case. Univariate analysis and logistic regression models were used for the analysis of data.

**Results:**

Two hundred and forty-nine patients (83 cases, 166 controls) were selected. Eighty-three episodes of major infections were analyzed; *E. coli*, *S. aureus*, *M. tuberculosis *and *S. pneumoniae *being the most frequent isolates. Univariate analysis identified several variables related with infection: lung and renal involvement, at or previous to the study point; leukopenia at the study point; antiphospholipid antibody-positivity and treatment with prednisone within 3 months previous to the study point, and the dose of prednisone received. Treatment with antimalarials, on the other hand, showed a strong inverse association with major infections. Logistic regression models identified treatment with antimalarials (odds ratio (OR) = 0.06, 95% confidence interval (CI) = 0.02 to 0.18), prednisone dose (OR = 1.12, 95% CI = 1.04 to 1.19) and lung involvement (OR = 4.41, 95% CI = 1.06 to 18.36) as significant and independent predictors of major infections. No significant interactions among these three variables were found. Further adjustment for potential confounders related with antimalarial treatment did not change the results.

**Conclusions:**

The risk of major infections in patients with SLE is mostly influenced by treatment. Prednisone treatment, even at moderate doses, increases the risk, whilst antimalarials have a protective effect.

## Introduction

Infections are among the most important causes of morbidity and mortality in patients with systemic lupus erythematosus (SLE) [1]. Several studies have analyzed the prevalence and associated clinical and laboratory features of infection in SLE. Disease duration, disease activity, leukopenia, steroids and immunosuppressive drugs have been linked, in different combinations, to an increased risk of infection [2-9]. The definitions of infection have been heterogeneous, however, with many studies also including minor infections with low prognostic implications. Moreover, while the role of prednisone, cyclophosphamide and other immunosuppressive drugs has been extensively analyzed, few studies included antimalarials among the variables potentially influencing the risk of infection.

The aim of the present study is to identify the potential predictors of infections in a prospective observational cohort of patients with SLE, limiting the analysis to major episodes with important clinical impact and including antimalarials among the potential variables influencing the final outcome.

## Materials and methods

### Study design and patients

A nested case–control study design was used within the prospective Lupus-Cruces cohort. The local institutional review board approved the study protocol, without the need for informed consent (study code CEIC E08/21), in compliance with the Helsinki Declaration.

All patients fulfilling the updated American College of Rheumatology criteria for the classification of SLE [10] on attending the Internal Medicine Department, Hospital de Cruces – a tertiary teaching center associated with the University of the Basque Country – have been included in the ongoing prospective, observational study. Patient data since 1973 have been included in the database. Patients on active follow-up give informed consent to include their data in our records. The time of inclusion in the cohort was the point when four American College of Rheumatology criteria were first met. This was also the time zero of follow-up.

Patients are regularly assessed every 3 months, although the course of the disease may modify this schedule. Clinical and immunological variables, including current treatments and occurrence of major complications, such as infections, are recorded in a standardized protocol and incorporated into the database at every follow-up visit. The Systemic Lupus International Collaborating Clinics Damage Index (SDI) has been regularly used to quantify the presence of irreversible organ damage [11]. The Systemic Lupus Erythematosus Disease Activity Index (SLEDAI) 2 K was used to measure lupus activity [12]. Antimicrobial prophylaxis has not been used in our cohort. Likewise, regular pneumococcal vaccination has been indicated only recently.

### Study endpoint

The endpoint of the present study is the occurrence of major infections, defined as those that are disseminated (septicemia), affecting deep organs (pneumonia, pyelonephritis, endocarditis, meningitis), requiring hospital admission for treatment (severe soft tissue infection, disseminated Varicella zoster) or causing death. The diagnosis of infection was always made by our team according to clinical, microbiological and imaging criteria.

### Statistical analysis

To overcome a potential immortal treatment bias – that is, the decreased possibility of patients with an early final event receiving the study drug, thus spuriously increasing its protective effect [13] – we identified patients with major infections (cases) and selected two control individuals (without any major infection) per case, matched for the time of follow-up until the event (± 1 year) and the age at diagnosis. Baseline data for the whole cohort and for the sample included in the case–control study were analyzed in order to prevent a selection bias.

The frequency, type and causal agent (if known) of major infections were described. Potential predictors of infection included variables with a possible relation to infection in previous studies, markers of SLE activity and irreversible organ damage and variables with a known prognostic influence in SLE: sex, treatment with antimalarials, prednisone, azathioprine, cyclophosphamide, methotrexate, mycophenolate, cyclosporine or any immunosuppressive drug within 3 months previous to the study point; dose of prednisone (mg/day) at the study point; active lupus nephritis, lung involvement (defined as either active disease or residual restrictive damage) and thrombocytopenia at any time before and at the study point; hypoclomplementemia C3/C4 and leukopenia at the study point; presence of anti-DNA and antiphospholipid antibodies (aPL) according to Sapporo criteria [14] at any time before the study point; SLEDAI at SLE diagnosis and at the study point; and the SDI at the first 6 months after the diagnosis of SLE. Age at diagnosis of SLE was excluded from the analysis due to the age-matched case–control design of the study. The study point was defined as the moment of suffering the first major infection in the cases and the last visit of follow-up in the controls.

The chi-squared test with Yates' correction, the Student *t *test and the Mann–Whitney U test were used for statistical comparisons of categorical, normally and non-normally distributed variables, respectively. In order to identify the independent predictors of major infections, all variables with *P *< 0.20 in the univariate analysis were entered into a binary logistic regression model with sequential elimination of nonsignificant variables. Interactions between the variables in the final model were tested and those resulting significant were added as further adjustment variables. The goodness of the final model was tested by calculating the area under the receiver operating characteristic curve. All of the statistical calculations were carried out using the statistical software SPSS 11.0.4 for Mac OS X (SPSS Inc., Chicago, IL, USA).

## Results

### Demographic data

Two hundred and eighty-four patients were included in the cohort at the time of this study. Two hundred and fifty patients (88%) were women and 282 (99%) were white. The mean (standard deviation) age at diagnosis was 36 (16) years. One hundred and ninety-seven patients (70%) were ever treated with antimalarials, 238 (84%) with prednisone and 126 (44%) with immunosuppressive drugs. Two hundred and ten patients (74%) had no early damage, 66 patients (23%) had a SDI of 1 to 2 and eight patients (3%) had SDI >2.

Two hundred and forty-nine patients (83 cases and 166 controls) were selected from the cohort for inclusion in the nested case–control analysis. Two hundred and twenty-three patients (90%) were women and 247 (99%) were white. The mean (SD) age at diagnosis was 36.2 (18) years for cases and 36.6 (16) years for controls (*P *= 0.84). The mean (SD) follow-up was 8.33 (7) years and 9.7 (7) years, respectively (*P *= 0.13). One hundred and seventy-six patients (70%) were ever treated with antimalarials, 208 (83%) with prednisone and 116 (46%) with immunosuppressive drugs. One hundred and eighty-one patients (72%) had a SDI of 0, 60 patients (24%) had a SDI of 1 to 2 and seven patients (3%) had SDI >2. The subgroup selected for the case–control study was thus fully representative of the whole cohort.

### Frequency and types of major infections

Eighty-three patients (29% of the cohort) suffered at least one major infection. Fifty-five patients (66%) suffered one infection, 22 patients (27%) suffered two infections, five patients (6%) suffered three infections and one patient suffered nine major infections. The 83 infections analyzed in this study are detailed in Table 1. Eleven of these 83 infections (13%) were nosocomial.

**Table 1:** Frequency of major infections (n = 83)

	Number of cases	%
Pneumonia	34	41
Bacteremia	20	24
Cellulitis and skin abscess	8	10
Tuberculosis	6	7
Meningitis–encephalitis	5	6
Pyelonephritis	4	5
Abdominal infections	3	4
Esophageal candidiasis	1	1
Osteomyelitis	1	1
Disseminated Varicella	1	1

The causal agent of infection was established in 43 cases (Table 2). *Escherichia coli*, *Staphylococcus aureus*, *Mycobacterium tuberculosis *and *Streptococcus pneumoniae *were the most frequent isolates. No cases of *Pneumocystis jivorecii *(formerly *Pneumocystis carinii*) were seen in our cohort, despite the fact that prophylaxis with cotrimoxazole has not been used. Four patients received pneumococcal vaccine, none of whom had a major infection.

**Table 2:** Causal agents of major infections

Microorganism	Number of cases	%
*Escherichia coli*	7^a^	16
*Staphylococcus aureus*	6^a^	14
*Mycobacterium tuberculosis*	6	14
*Streptococcus pneumoniae*	5	12
Salmonella	4	9
*Psuedomonas aeruginosa*	3	7
*Candida albicans*	3	7
*Neisseria meningitides*	2	5
Varicella Zoster virus	2	5
Campylobacter	1	2
Legionella	1	2
Herpes simplex virus	1	2
Serratia	1	2
*Staphylococcus epidermidis*	1	2
Aspergillus	1	2

^a^One patient suffered a combined septicemia by *E. coli *and *S. aureus*.

Eight patients died as a consequence of infection: pneumonia was the cause of death in four cases, bacteremia with septic shock in three cases and peritonitis in one case.

### Predictors of major infection

Results of univariate analysis are presented in Table 3. Several clinical, immunological and therapeutic variables were associated with major infections: lung and renal involvement at or previous to the study point, leukopenia at the study point, aPL-positivity and treatment with prednisone within 3 months previous to the study point. The dose of prednisone received was also related to the risk of infection. Treatment with antimalarials, on the other hand, showed a strong inverse association with major infections. Treatment with any immunosuppressive drug or with each of the individual agents (azathioprine, methotrexate, cyclophosphamide, mycophenolate or cyclosporine) did not confer a higher risk of suffering a major infection. Likewise, the presence of early damage was not associated with the occurrence of major infections.

**Table 3:** Univariate analysis

	Major infection (n = 83)	No major infection (n = 166)	*P *value
Female sex	73/83 (88)	150/166 (90)	0.70
Nephritis at study point	15/83 (18)	10/166 (6)	<0.01
Previous nephritis	34/83 (41)	39/166 (23)	<0.01
Thrombocytopenia at study point	6/83 (7)	7/166 (4)	0.50
Previous thrombocytopenia	19/83 (23)	27/166 (16)	0.27
Lung disease at study point	12/83 (14)	6/166 (4)	<0.01
Previous lung disease	13/83 (15)	8/166 (5)	<0.01
Leukopenia at study point	32/83 (39)	23/166 (14)	<0.01
Low complement at study point	36/81 (44)	52/163 (32)	0.08
Anti-DNA antibodies	61/83 (73)	102/166 (61)	0.08
Antiphospholipid antibodies	43/83 (52)	57/166 (34)	0.01
SDI = 0	57/83 (69)	125/166 (75)	
SDI = 1 to 2	22/83 (26)	38/166 (23)	0.29
SDI >2	4/83 (5)	3/166 (2)	
SLEDAI at diagnosis	10 (1 to 25)	9 (1 to 22)	0.23
SLEDAI at study point	4 (0 to 24)	3 (0 to 21)	0.15
SLEDAI at diagnosis >12	25/81 (31)	33/163 (20)	0.09
SLEDAI at study point >12	5/81 (6)	7/163 (4)	0.74
Antimalarials	18/83 (22)	128/166 (77)	<0.01
Months on antimalarials	0 (0 to 300)	24 (0 to 192)	<0.01
Azathioprine	16/83 (19)^a^	20/166 (12)	0.18
Methotrexate	2/83 (3)	13/166 (8)	0.12
Cyclophosphamide	3/83 (4)	2/166 (1)	0.42
Mycophenolate	1/83 (1)	5/166 (3)	0.66
Cyclosporine	3/83 (2)^a^	4/166 (5)	0.34
Any immunosuppressor	23/83 (30)	44/166 (26)	0.96
Prednisone	62/83 (75)	90/166 (54)	<0.01
Prednisone dose (mg/day)	7.5 (0 to 90)	2.5 (0 to 60)	<0.01

Data presented as *n*/*N *(%) or median (range). SDI, Systemic Lupus International Collaborating Clinics Damage Index; SLEDAI, Systemic Lupus Erythematosus Disease Activity Index. ^a^Two patients were taking combined treatment with azathioprine and cyclosporine.

The logistic regression analysis largely reduced the number of predictors of major infections. Lung involvement at the study point (odds ratio (OR) = 3.90, 95% confidence interval (95% CI) = 0.99 to 15.2), aPL positivity (OR = 2.50, 95% CI = 1.25 to 5.01) and the dose of prednisone at the study point (OR = 1.11, 95% CI = 1.05 to 1.17) increased the risk of infection, whilst antimalarials retained their protective effects against infection after adjustment (OR = 0.09, 95% CI = 0.05 to 0.18). The remaining independent variables entered into the regression did not show a significant independent effect. The ORs for the significant variables did not differ between the initial model and the final model: 2.25 and 3.90 for lung involvement, 2.21 and 2.50 for aPL, 1.10 and 1.11 for prednisone dose, and 0.09 and 0.09 for antimalarials, respectively.

The interactions between the significant independent variables were tested. Only interactions between antimalarials and aPL (*P *= 0.016) and between the dose of prednisone and aPL (*P *= 0.001) were significant. When these interactions were entered into the final logistic regression model, aPL was no longer significant. The other three variables retained the degree of statistical significance without relevant modifications in the magnitude of their effect on major infections (see Table 4). The area under the receiver operating characteristic curve of this model was 0.88 (95% CI = 0.83 to 0.92). The inclusion of the continuous variable of time (months) on antimalarials instead of the categorical variable of treatment with antimalarials rendered similar results in the final model (OR = 0.991, 95% CI to 0.984 to 0.999, *P *= 0.018).

**Table 4:** Logistic regression, final model (dependent variable, major infection)

Variable	Odds ratio	95% confidence interval
Lung involvement at study point	4.41	1.06 to 18.36
Prednisone dose (mg/day)	1.12	1.04 to 1.19
Antimalarials	0.06	0.02 to 0.18
Antiphospholipid antibodies	1.88	0.66 to 5.32
Antiphospholipid antibodies × antimalarials	2.21	0.52 to 9.33
Antiphospholipid antibodies × prednisone dose	0.98	0.88 to 1.11

Antiphospholipid antibodies × antimalarials and antiphospholipid antibodies × prednisone dose are interaction variables.

### Influence of disease severity on antimalarial treatment and its effect on major infections

Several variables related with disease severity were compared among antimalarial users and nonusers. Patients with antimalarial treatment were younger at diagnosis, less likely to have suffered nephritis and leukopenia, and were receiving mycophenolate mofetil in a lower proportion at the study point. Likewise, they had accrued less early damage than antimalarial nonusers (Table 5). When these variables were added to the logistic regression final model, the adjusted OR of suffering a major infection for antimalarial users was 0.070 (95% CI = 0.030 to 0.155), thus remaining largely unchanged (Table 6).

**Table 5:** Clinical characteristics according to treatment with antimalarials at the study point

	Antimalarials		*P *value
		
	Yes (n = 146)	No (n = 103)	
Age at diagnosis (years)	34 (14)	40 (18)	<0.01
Female sex	131/146 (89)	63/103 (86)	1.00
Nephritis ever	35/146 (24)	50/103 (48)	<0.01
Lung involvement ever	11/146 (7.5)	13/103 (12.6)	0.26
Thrombocytopenia ever	24/146 (16)	17/103 (26)	0.08
Leukopenia ever	94/146 (64)	85/103 (82)	<0.01
Anti-DNA antibodies ever	96/146 (66)	67/103 (65)	1.00
Low complement at study point	48/143 (34)	40/101 (40)	0.40
Prednisone at study point	89/146 (61)	63/103 (62)	1.00
Azathioprine at study point	18/146 (12)	18/103 (18)	0.34
Cyclophosphamide at study point	2/146 (1)	3/103 (3)	0.69
Methotrexate at study point	12/146 (8)	3/103 (3)	0.144
Mycophenolate at study point	6/146 (45)	0/103 (0)	0.04
SDI = 0	121/146 (83)	61/103 (59)	
SDI = 1 to 2	25/146 (17)	35/103 (34)	0.001
SDI >2	0/146 (0)	7/103 (7)	
SLEDAI at diagnosis	9 (1 to 22)	9 (2 to 25)	0.94
SLEDAI at study point	4 (0 to 21)	3 (0 to 24)	0.84
SLEDAI at diagnosis >12	33/143 (23)	25/101 (25)	0.88
SLEDAI at study point >12	6/143 (4)	6/101(6)	0.75

Data presented as mean (standard deviation), *n*/*N *(%) or median (range). SDI, Systemic Lupus International Collaborating Clinics Damage Index; SLEDAI, Systemic Lupus Erythematosus Disease Activity Index.

**Table 6:** Logistic regression, adjustment for confounders related with antimalarial treatment (dependent variable, major infection)

Variable	Odds ratio	95% confidence interval
Lung involvement at study point	3.11	0.79 to 12.31
Prednisone dose (mg/day)	1.10	1.04 to 1.17
Antimalarials	0.07	0.03 to 0.15
Age at diagnosis	0.99	0.97 to 1.01
Leukopenia ever	1.99	0.82 to 4.82
Nephritis ever	1.61	0.72 to 3.64
Mycophenolate at study point	1.52	0.15 to 15.40
SDI = 0	Reference	
SDI = 1 to 2	0.42	0.18 to 1.00
SDI > 2	0.22	0.03 to 1.65

SDI, Systemic Lupus International Collaborating Clinics Damage Index.

## Discussion

Our study showed that one-third of lupus patients developed serious infections during follow-up. Common bacteria, such as *E. coli*, Staphylococcus and *S. pneumoniae *were the most frequent isolates. Several clinical, immunological and therapeutic variables showed association with the risk of suffering major infections. Only three variables, however, had a significant independent effect: lung disease, dose of prednisone and treatment with antimalarials at the study point, the latter showing a strongly protective effect.

Clinical features predisposing SLE patients to infection are not well established. Immunosuppressive drugs have increased the frequency of infections in only a small proportion of studies [4,8,9]. Treatment with steroids has been identified as a risk factor in several studies [4,6,8,9], but not in all series [2,3,5,7]. Renal disease, despite being associated with infections in the univariate analysis in some series, has not retained the statistical significance in the multivariant analysis [5,6]. Only the series of the Hopkins Lupus Cohort found an independent effect of serum creatinine on the rate of hospitalizations due to infection [7]. Likewise, low complement levels have been significant independent predictors in three studies [7,8,15]. A SLEDAI score higher than 12 at diagnosis has also been found to increase the risk of infection in one recent study [15].

It is thus a difficult task to identify lupus patients at high risk of serious infectious complications. The inclusion in some studies of patients with both minor and major infections [3,6,9] complicates the interpretation of results. The introduction of ever variables and at the time of infection variables, depending on the studies, also adds noise to the conclusions. In addition, many variables (lupus activity, renal disease, low complement, treatment with steroids and immunosuppressive drugs) are intimately related with each other in clinical practice, and thus their relative weight and interactions are difficult to ascertain, even with multivariate analysis.

Our study could somewhat clarify the clinical profile of patients prone to suffering serious infections with the potential to influence the prognosis of SLE. The nested case–control design, matched for age and time to event, reduces the possibility of an immortal treatment bias [12]. We analyzed clinical and immunological variables that could have an effect on immunity (leukopenia, lupus nephritis, drug therapy, complement levels), could reflect lupus activity (SLEDAI score, anti-DNA antibodies, lupus nephritis, lung disease, leukopenia, thrombocytopenia, drug therapy) and/or have known prognostic significance (sex, aPL, lupus nephritis, lung involvement, leukopenia, thrombocytopenia, early damage). Treatment variables were recorded in close temporal relationship with the study point.

In the present study, the prednisone dose at the time of the event had a facilitating effect on infections, in line with data coming from other series [4,6,8,9]. It is noteworthy that the median dose of patients with major infections was only 7.5 mg/day. According to the results of the logistic regression, each increase of 10 mg/day prednisone multiplied by 11 the risk of suffering a serious infection. Every effort should therefore be made to limit both the dose and the time of exposure to steroids of lupus patients, also taking into account the close relation of irreversible damage and prednisone use [16]. On the contrary, no individual immunosuppressive drug or treatment with any of them increased the risk for serious infections. This apparent paradox could be explained by the low numbers of patients on cyclophosphamide, which is usually given in our unit as low-dose pulses, with a lesser frequency of infectious complications [17]. We also excluded minor infections, such as nondisseminated herpes zoster and minor urinary tract infections, that have been associated with immunosuppressive drugs like azathioprine, cyclophosphamide and methotrexate [18,19]. The association between lung disease and infection in our cohort could be explained by the frequent occurrence of serious respiratory infections in patients with restrictive disease (six out of 14 patients). Noteworthy, complement levels or the SLEDAI score did not modify the risk of major infection of our patients [15].

The most relevant finding of the present study, however, was that patients taking antimalarials were 16 times less likely to suffer a major infection. From the pharmacological point of view, this effect is not surprising. Originally synthesized as antiparasitic agents, these drugs have a wide range of antimicrobial effects [20]. *In vitro *activity against bacteria (*Tropheryma whipplei*, *S. aureus*, *Legionella pneumophila*, *Francisella tularensis*, Mycobacterium spp., *Salmonella typhi*, *E. coli*, *Borrelia burgdorferi*), fungi (*Histoplasma capsulatum*, *Cryptococcus neoformans*, *Aspergillus fumigatus*) and viruses (including human immunodeficiency virus) has been reported [20]. Antimalarials interfere with invasion and internalization of *E. coli *into host cells [21]. Clinical studies have established the combination doxycycline plus hydroxychloroquine (HCQ) as the standard treatment for chronic Q fever [20]. Likewise, combination treatment with HCQ, OH carbamide and didanosine reduces the human immunodeficiency virus load, having been proposed as a cheaper alternative for the treatment of AIDS [22].

The antibacterial effects of antimalarials are mediated by pH-dependent iron deprivation and by increasing the phagolysosomal pH, both inhibiting the growth of intracellular organisms [20]. Likewise, the antiviral effects are related to increasing the lysosomal pH, which disrupts hydrolases and inhibit the post-translational modification of newly synthesized proteins [23].

Two previous studies in patients with lupus suggest the protective effect of antimalarials against infection. Sisó and colleagues, in a retrospective cohort study of patients with lupus nephritis, found a lower frequency of infections among those previously treated with antimalarials [24]. Bultink and colleagues, in a study designed to analyze the effect of the deficiency of functional mannose-binding lectin, found that treatment with HCQ was protective of major infections, with an adjusted OR (OR = 0.05, 95% CI = 0.01 to 0.23) very similar to that obtained in the present study [5]. The authors attributed this association, however, to the presumed lesser severity of lupus in patients taking HCQ. In addition, in 1996 Podrebarac and colleagues reported two cases of *P. carinii *pneumonia in patients with SLE treated with high-dose steroids shortly after discontinuation of HCQ [25]. We believe that our study confirms the antimicrobial effects of antimalarials, which can be actually an additional reason for the increased survival seen in SLE patients taking these drugs [24,26,27].

Our study has the main limitation of the observational design – the specific treatment of each patient was therefore not randomized, but rather decided on clinical grounds. In fact, patients given antimalarials were younger and had less severe disease, with a lower frequency of nephritis, leukopenia and early irreversible damage. When these adjustment variables were added to the regression model, however, the protective effect of antimalarials on the occurrence of major infections remained unchanged.

## Conclusions

The treatment received by the patient is the most important determinant of the risk of serious infections. Prednisone should therefore be used with caution, and maintenance doses above 5 mg/day are best avoided. In addition, treatment with HCQ would be indicated in all patients with SLE without contraindications, given the excellent safety profile and the wide range of long-term beneficial effects, including the potential protection from serious infections [28].

## Abbreviations

aPL: antiphospholipid antibodies; CI: confidence interval; HCQ: hydroxychloroquine; OR: odds ratio; SDI: Systemic Lupus International Collaborating Clinics Damage Index; SLE: systemic lupus erythematosus; SLEDAI: Systemic Lupus Erythematosus Disease Activity Index.

## Competing interests

The authors declare that they have no competing interests.

## Authors' contributions

GR-I conceived of the study, participated in the design and coordination of the study, performed the statistical analysis and prepared the manuscript. NO participated in the design of the study and in the acquisition of data. IR-A participated in the acquisition of data and helped to draft the manuscript. AM-B helped to draft the manuscript. M-VE participated in the design of the study and helped to draft the manuscript. CA participated in the conception of the study. All authors read and approved the final manuscript.
